# Microsatellite/SSR dataset: characterization of apple cultivars of the German Fruit Genebank

**DOI:** 10.1038/s41597-025-04390-5

**Published:** 2025-01-15

**Authors:** Lea Broschewitz, Stefanie Reim, Henryk Flachowsky, Monika Höfer

**Affiliations:** Julius Kühn Institute (JKI), Federal Research Centre for Cultivated Plants, Institute for Breeding Research on Fruit Crops, Pillnitzer Platz 3a, 01326 Dresden-Pillnitz, Germany

**Keywords:** Plant genetics, Plant genetics, Genetic markers, Conservation biology, Plant molecular biology

## Abstract

The German Fruit Genebank is a decentralized network focused on coordinating various germplasm collections across Germany to conserve and utilize the genetic resources of native fruit species. This aim emphasizes the necessity of trueness-to-type validation of genetic resources based on pomological and molecular characteristics. Between 2009 and 2021, multiple projects were undertaken to create an inventory of the apple (*Malus* ssp.) network of the German Fruit Genebank. Eight partner locations across Germany participated in these projects to assess the authenticity of apple cultivars. Firstly, at least two knowledgeable experts from the German Pomological Association characterized the apple accessions pomologically. Secondly, DNA fingerprint analysis was conducted for molecular characterization. The DNA fingerprint analysis employed a set of 17 simple sequence repeat markers and 8 reference genotypes recommended by the European Cooperative Programme for Plant Genetic Resources *Malus/Pyrus* working group and was carried out by ecogenics GmbH (c/o Microsynth AG, Balgach, Switzerland). 8,184 samples were analysed, resulting in the compilation of 1,404 molecular groups that correspond to distinct apple cultivars.

## Background & Summary

The German Fruit Genebank (GFG; https://www.deutsche-genbank-obst.de/) is a decentralised genebank network for fruit genetic resources in Germany. Its goal is the effective and long-term conservation and utilization of genetic resources for research and breeding. Furthermore, it provides information about pomological and landscaping traits. The focus is put on cultivars of native fruit species, i.e. apple, prioritising German cultivars and selections, cultivars with socio-cultural, local or historic relation to Germany and cultivars with important pomological traits. Per fruit species a network is established, i.e. the German apple network. In line with the goals of the GFG, an inventory was conducted with the purpose of evaluating the authenticity and diversity of apple cultivars resulting in the presented dataset^1^.

The authenticity evaluation of the cultivars is guaranteed in a two-step process. First, at least two experts of the German Pomological Association (Pomologen-Verein e.V., Hamburg, Germany, https://www.pomologen-verein.de/) did a pomological characterization to identify the true cultivar name of individual trees. Second, molecular analysis supplemented the results of the pomological characterization. 17 simple sequence repeat (SSR) markers, recommended by the European Cooperative Programme for Plant Genetic Resources (ECPGR; http://www.ecpgr.cgiar.org/) Working group *Malus/ Pyrus* were used to create individual genetic profiles per tree^2^. The genetic profiles were assigned into molecular groups based on similarity to one another. The pomological and molecular data were combined to ensure that all trees or samples sharing the same identified cultivar name are also included in the corresponding molecular group. The raw data consisted of 8,354 molecular samples and 170 samples were discarded during manual quality control and review in reference to the pomological information. Therefore, the data used for the compilation of consensus genetic profiles consisted of 8,184 samples in total. The 8,184 samples correspond to 7,890 apple trees from which leaf material was taken and tested, at times in repetition. Lastly, this data was compiled from several samples with individual genetic profiles to one consensus genetic profile per cultivar. 1,404 consensus genetic profiles were identified.

## Methods

### Data collection

On behalf of the Federal Ministry of Food and Agriculture, the Federal Office for Agriculture and Food awards contracts for surveys, inventories and non-scientific studies in the field of biodiversity. Funding for five projects was granted in two blocks from 2009 to 2014 (projects 2009–2014) and from 2017 to 2021 (projects 2017–2021) to determine the trueness-to-type of cultivars in the apple network of the GFG (grant numbers: 2809BE006, 2809BE010, 2813BE001 2816BE007 and 2816BE008). The plant material for characterization was provided by eight different GFG apple network partners located across Germany. The exact location of the partner apple collections and in which projects the partners participated can be viewed in Supplementary Table S1. All of the collections are grown as *ex situ* orchards. In the projects 2009–2014, a total of 2,939 individual trees were tested. The projects 2017–2021 were performed on 4,951 individual trees. The total amount of individual tested trees is 7,890 based on the tree location information. During both projects, some trees were tested multiple times, resulting in a slightly higher sample count later on. 259 trees were multi-tested between two and five times, leading to 8,354 molecular samples in the initial, raw dataset and 8,184 samples in the revised dataset used for analysis later on. The apple trees were visited on-site for pomological evaluation and samples from the respective trees were taken for molecular analysis. For the projects 2009–2014, the GFG-partner Competence Centre of Fruit Production - Lake Constance (CCFP, Bavendorf, Germany) supervised and coordinated the sampling. The plant samples consisted of clean and young leaf material, preferably shoot tips with five to six leaves. When there were no shoot tips available, five to six individual leaves were collected. The samples were taken from June to July 2011 at the different partner location (Supplementary Table S2). After collection, the samples were transferred to CCFP for storage at −18 °C until further analysis. During projects 2017–2021, the gathering of samples was done blindly by ecogenics GmbH (c/o Microsynth AG, Balgach, Switzerland) following a provided list with the location details of each tree. To minimize sampling errors, a system was established where the sampled tree, the sample envelope and the list were marked with the barcode.

### Pomological characterization

During the projects 2009–2014, the fruit samples of the six partner locations were used (Supplementary Table 1)^3^. All fruit samples were gathered at the location of CCFP for a central assessment. For each location, there was a plan of the orchard with row and tree numbers as well as a supposed cultivar name. In dependence of the ripening time of the respective cultivar, the fruits were collected locally and stored at low temperature. In beginning of November in the years 2009 and 2010, the fruits were moved to CCFP for storage until subsequent pomological determination. Eight pomologists who were part of the German Pomological Association and the Variety Preservation Centre Baden-Wuerttemberg (Bavendorf, Germany) conducted the pomological assessment. Namely, these were Michael Ruhnau, Hans-Thomas Bosch, Sabine Fortak, Jan Bade, Hans-Joachim Bannier, Dr. Werner Schuricht, Eckhart Fritz and Dr. Ulrich Mayr. For the pomological characterization, three to five healthy fruits were assessed as no fruit shows all characteristic traits at once. The fruits were evaluated for external traits such as shape, ground colour, hue, relative area, intensity or pattern of overcolour, number lenticels and area of russet^4^. Internal traits that were appraised include e.g. colour of flesh, core shape, kernels, vascular bundle, open or closed calyx. Finally, degustation characteristics such as firmness, texture, juiciness and flavour were assessed to determine the apple cultivar. A fruit sample and the respective tree was determined as a specific apple cultivar if at least two experts gave a consistent appraisal and no other experts intervened in the decision.

The pomological characterization of projects 2017–2021 was predominantly performed by Hans-Joachim Bannier and Dr. Werner Schuricht of the German Pomological Association^5^. The apple trees were evaluated on-site at the eight partner locations (Supplementary Table S1). Again, the pomologist was provided with plans of orchards and assumed cultivar names. This time, literature references used during the decision making for the cultivars had to be made available by the pomologist.

### Molecular characterization

In projects 2009–2014, plant samples were collected by Competence Centre of Fruit Production – Lake Constance and stored there until the analysis^6^. The samples were collectively sent to ecogenics GmbH (c/o Microsynth AG, Balgach, Switzerland) where they were processed^7^. During projects 2017–2021, the sample collection and subsequent molecular analysis was conducted by ecogenics GmbH (c/o Microsynth AG, Balgach, Switzerland)^8^. The used genotype references and SSR markers were recommended by the ECPGR Working group *Malus/Pyrus*^2^. The chosen reference genotypes were ‘Delicious’, ‘Fiesta’, ‘Michelin’, ‘Coop 2’, ‘Worcester Parmäne’, ‘Malling 9’, ‘Malus × floribunda 821’ and ‘Malus × robusta 5’ provided by INRAE (National Research Institute for Agriculture, Food and the Environment, Paris, France) as DNA samples. In projects 2009–2014, each reference sample was analysed once, while it was analysed four times in projects 2017–2021. Also during projects 2017–2021, additional blank tests were performed where four random samples were replicated four times. Unless stated otherwise, the same procedures were used for projects 2009–2014 and projects 2017–2021^7,8^.

### DNA extraction

From the plant leaf material, a single sheet punch was taken for DNA isolation. In projects 2009–2014, DNA was extracted with alkaline lysis in presence of TWEEN 20, followed by pH neutralization. The isolated DNA was contained in 50 mM Tris pH8, 100 mM NaCl, 1% TWEEN 10 and 1 mM EDTA. For projects 2017–2021, “Hot Shot”-method was used. To the sheet punch, buffer A (0.1 M NaOH, 2% Tween20 in twice-distilled water) was given and incubated at 95 °C for 15 min. Then, buffer B (0.1 M HCl, 0.1 M Tris-HCl pH8.0, 0.05 M EDTA in twice-distilled water) was added. An aliquot of the DNA isolate was taken in a dilution of 1:50. The DNA isolates were stored in 96-well-plates. Each 96-well-plate included an isolation control (no leaf material), a PCR control (no DNA template), three multiplex-specific allele ladders and a positive control (freshly isolated leaf sample).

### Genetic analysis

For this genetic analysis, 17 simple sequence repeat (SSR) or microsatellite markers were used, namely CH01h01, CH01f07a, CH03d07, CH04c07, CH05f06, CH01f03b, GD12, CH02d08, CH05e03, CH02c09, CH02g09, CH01f02, CH04f10, COL, Hi02c07, CH04e05, GD147^9–11^. Primer sequences for the markers are available in Table 1 and the primers were provided by Microsynth AG (Balgach, Switzerland). Most of the used markers are reported to bind single locus, except GD12 for which it is unknown and Hi02c07 and Ch04e05 which are presumably multi locus binding^12^.

**Table 1:** Primer sequences of the used SSR markers.

Marker	Forward primer 5′-3′-sequence	Reverse primer 5′-3′-sequence
*CH01h01*	GAAAGACTTGCAGTGGGAGC	GGAGTGGGTTTGAGAAGGTT
*CH01f07a*	CCCTACACAGTTTCTCAACCC	CGTTTTTGGAGCGTAGGAAC
*CH03d07*	CAAATCAATGCAAAACTGTCA	GGCTTCTGGCCATGATTTTA
*CH04c07*	GGCCTTCCATGTCTCAGAAG	CCTCATGCCCTCCACTAACA
*CH05f06*	TTAGATCCGGTCACTCTCCACT	TGGAGGAAGACGAAGAAGAAAG
*CH01f03b*	GAGAAGCAAATGCAAAACCC	CTCCCCGGCTCCTATTCTAC
*GD12*	TTGAGGTGTTTCTCCCATTGGA	CTAACGAAGCCGCCATTTCTTT
*CH02d08*	TCCAAAATGGCGTACCTCTC	GCAGACACTCACTCACTATCTCTC
*CH05e03*	CGAATATTTTCACTCTGACTGGG	CAAGTTGTTGTACTGCTCCGAC
*CH02c09*	TTATGTACCAACTTTGCTAACCTC	AGAAGCAGCAGAGGAGGATG
*CH02g09*	TCAGACAGAAGAGGAACTGTATTTG	CAAACAAACCAGTACCGCAA
*CH01f02*	ACCACATTAGAGCAGTTGAGG	CTGGTTTGTTTTCCTCCAGC
*CH04f10*	GTAATGGAAATACAGTTTCACAA	TTAAATGCTTGGTGTGTTTTGC
*COL*	AGGAGAAAGGCGTTTACCTG	GACTCATTCTTCGTCGTCACTG
*Hi02c07*	AGAGCTACGGGGATCCAAAT	GTTTAAGCATCCCGATTGAAAGG
*CH04e05*	AGGCTAACAGAAATGTGGTTTG	ATGGCTCCTATTGCCATCAT
*GD147*	TCCCGCCATTTCTCTGC	GTTTAAACCGCTGCTGCTGAAC

This information was taken from the High-quality Disease Resistant Apples for a Sustainable Agriculture (HiDRAS) website (https://sites.unimi.it/camelot/hidras/HiDRAS-SSRdb/pages/index.php) and the primers were provided by Microsynth AG (Balgach, Switzerland).

For detection with capillary electrophoresis, fluorophores were attached at the 5′-end of the respective forward primer. The used fluorophores in this assay were fluorescein (FAM) as well as rhodamine dye ATTO532, ATTO550 and ATTO565. The resulting combinations of markers and fluorophores can be seen in Table 2.

**Table 2:** Three multiplex-PCR-admixtures.

Multiplex-PCR admixture	Microsatellite marker	Fluorophore
M1	CH01h01	FAM
	CH01f07a	FAM
	CH03d07	ATTO532
	CH04c07	ATTO550
	CH05f06	ATTO550
	CH01f03b	ATTO565
M2	GD12	FAM
	CH02d08	ATTO565
	CH05e03	ATTO550
	CH02c09	ATTO550
	CH02g09*	ATTO565*
	CH01f02*	ATTO532*
M3	CH04f10	FAM
	COL	ATTO532
	Hi02c07	ATTO550
	CH04e05	ATTO550
	GD147	ATTO565

Combinations of microsatellite marker and corresponding fluorophore are shown. The same combination were chosen for each project (2009–2014 & 2017–2021) except for CH02g09 and CH01f02. *In the first project 2009–2014 combination CH02g09_ATTO532 and CH01f02_ATTO565 were used while CH02g09_ATTO565 and CH01f02_ATTO532 were used in the second project 2017–2021 but this had no influence in the molecular analysis.

A Multiplex-PCR-Kit (Qiagen, Germany) was used with the thermocycler program with an initial denaturation cycle at 95 °C for 10 min followed by 40 cycles of denaturation at 94 °C for 0.5 min, primer hybridization at 55 °C for 1.5 min and elongation at 72 °C for 1 min. The PCR reaction was ended with a final elongation cycle at 72 °C for 30 min.

The fragment length analysis was performed on an 3730XL DNA Analyzer (Applied Biosystems, Thermo Fisher Scientific Inc., Waltham, MA, USA) with following conditions: 10 s injection time, 1.6 kV injection current, 2100 s run time, 15 kV run current, 50 cm capillary length, POP7 Polymer, Dye set G5 filter. Dye size standard ECO500 (ecogenics GmbH, c/o Microsynth AG, Balgach, Switzerland) modified with Dyomics 630 fluorophore and GeneScan™ 500 LIZ™ (Applied Biosystems, Thermo Fisher Scientific Inc., Waltham, MA, USA) were used in projects 2009–2014 and projects 2017–2021, respectively.

### Clustering of genetic profiles into gene groups

The SSR marker specific chromatograms resulting from the fragment length analysis were analysed with GeneMarker software V2.6.4 (SoftGenetics LLC., State College, PA, USA). In project 2009–2014 raw allele data of the chromatograms was provided and further examined by Dr. Juliane Würdig and André Kroll (data unpublished, JKI-ZO Dresden-Pillnitz)^13^. A program (unpublished, JKI-ZO Dresden-Pillnitz) was develop to distinguish and group identical genetic profiles without error margin. For each difference in a genetic profile, a new group was built with i.e. MD_0001 as an identifier. Here, MD is an abbreviation for *Malus* x *domestica* followed by an underscore and a four-decimal, continuous number.

During projects 2017–2021, this process was performed and adjusted by ecogenics GmbH (c/o Microsynth AG, Balgach, Switzerland)^8^. Between all available samples, including projects 2009–2014, the pairwise distance was calculated (Equation 1). To adjust for technical differentiations in allele fragment lengths (AFLs), a difference of ±1 bp was allowed. Pairwise distances between individual samples were calculated through an internally developed script for Python^14^, based on the equation for the Dice-Sørensen coefficient^15,16^:

1 $${pairwise\; distance}=1-\left(\frac{2\times \sum {common\; alleles}}{\sum {alleles\; sample}1+\sum {alleles\; sample}2}\right)$$

Then, the pairwise distances were clustered into gene groups again using the same identifies as before. A 25% cut-off in allele data difference between groups was determined based on the distribution of pairwise distances. Therefore, genetic profiles that are 75 to 100% identical group together. This step was performed in R^17^. This calculation and the inclusion of the dataset of projects 2009–2014 data set lead to the merge of some previous gene groups i.e. MD_0002/MD_0003. Some samples of projects 2009–2014 were additionally analysed and aligned with international data for assigning the Malus UNiQue genotype code (MUNQ) by INRAE (National Research Institute for Agriculture, Food and the Environment, Paris, France)^18,19^.

### Trueness-to-type criteria

The trueness-to-type criteria were established for use by the GFG to help express the authenticity of a given cultivar. The system was assembled based on the pomological and molecular results on a scale from “0” to “5” and “R” (Table 3). The criteria were originally given by the pomological experts based on their assessment. Later, the criteria per cultivar were confirmed and adjusted as needed with the additional knowledge provided by the molecular analysis. On one hand, cultivars are judged to be true-to-type if they scored a “1”, “2”, “5”. Reference samples (“R”) are also true-to-type and during analyses and on the GFG website, “R” is substituted by “1”. With these cultivars, the evaluation was straightforward and there was little to no ambiguity between i.e. literature references and the assessed fruit. On the other hand, cultivars evaluated with a “0”, “3” or “4” are deemed not true-to-type for the moment due to different resons. Cultivars with “0” have unique cultivar names but the trees deceased during the projects and the assessment could not be completed. The cultivar could still be within the GFG collection e.g. located at a different partner, but not yet tested, and therefore the cultivar was not removed yet. Not determined cultivars (“3”) might be authentic cultivars but the pomological assessment could not classify them as such because of e.g. missing literature (historical) reference and missing information on newly bred, local or foreign cultivars. The evaluation with a “4” indicates that the cultivar could not be assessed pomologically due to no or unsuitable fruit development. All these three evaluation scores therefore represent cultivars that are not true-to-type. These criteria can be adjusted in the future e.g. by a new (pomological) assessment.

**Table 3:** Explanation for trueness-to-type criteria as used in the German Fruit Genebank (GFG).

trueness-to-type GFG criteria		explanation
0	deceased	tree deceased during project duration
1	true-to-type	pomologically and molecularly assessed
2	true-to-type (group/mutant)	pomologically and molecularly assessed
3	not determined	no references available, cultivar name unknown
4	not assessed	not assessed (pomologically)
5	true-to-type with reservations	pomologically assessed, not assessed molecularly
		molecularly assessed for at least 3 accessions and pomologically assessed for at least 2 accessions
		pomologically assessed with reservation, molecularly assessed
R	Reference	Reference cultivar based on ECPGR guidelines (true-to-type)

### Identification of unique genetic profiles per apple cultivar

#### Manual inspection and adjustments of the data set

The raw data set of combined projects 2009–2014 and 2017–2021 consisted of 8,354 samples with complete pomological and molecular data (unpublished, JKI-ZO Dresden-Pillnitz). The aim was to define a unique genetic profile for each apple cultivar from the totality of samples. To achieve this, it was aimed to achieve consensus between the molecularly determined molecular groups and the pomologically determined cultivars. That means that all samples of the same cultivar that were assessed should molecularly group together as well. No molecular differentiation could be made between original cultivars and their mutations while the pomologists were able to distinguish them. Mutations of cultivars are also classified as independent cultivars but this could not be represented when deciding for a unique genetic profile by using SSR markers.

Individual samples that did not show a correspondence between cultivar name and gene group (with the exception of respective mutations) had to manually examined and possibly be corrected or removed. The main reasons for the lack of a correspondence were swapped samples, where the molecular sample was probably taken from a different tree than intended. In these cases, the sample was not grouping with other individuals of the same pomologically determined cultivar but with a completely different cultivar. Or, due to technical errors in the PCR, incorrect molecular groups were formed and deviating genetic profiles were observed as result. Here, it was prominent that only one or two individual samples create a new molecular group under a pre-existing cultivar name. Additionally, the corresponding second tree of the same accession would group with the correct, pre-existing cultivar. This circumstance with addition to visual assessment of the genetic profiles lead to the assumption of technical errors. These samples were either annotated to the correct gene group of the pre-existing cultivar or completely removed. Also, samples and trees which where pomologically determined as rootstocks, crab apples or wild apples were removed as they are not supposed to be part of the collection of the GFG.

Another manual adjustment of the molecular analysis was the splitting of the determined gene groups based on the pomological information and assessment of the genetic profiles. In cases of closely related di- and triploid cultivars, the used 25% range of the pairwise distance allowed these cultivars to group together when in fact they are different cultivars.

An additional modification of the molecular data was done directly to the allele fragments lengths of SSR marker CH02g09. During visual assessment it was noticed, that CH02g09 showed shifts of the reported AFLs of 2 bps between projects 2009–2014 and projects 2017–2021. This was predominantly apparent at AFLs between 122 bp and 140 bp. If this shift was observed at individual genetic profiles of the same gene group, the values found in projects 2009–2014 were adjusted to respective values of projects 2017–2021. This modification was performed in agreement and after consultation with ecogenics GmbH (c/o Microsynth AG, Balgach, Switzerland) who had access to the chromatograms.

The pomological information also needed revision in some cases. The most predominant revision was the unification of the cultivar name by i.e. correcting spelling errors, using the preferred cultivar name instead of a synonym or translating names of foreign language to German. Another important point was the handling of cultivars that could not be determined due to insufficient references or fruit samples. In these cases, the original cultivar name as provided by the holder of the collection was used unless the experts previously rejected this identity or the cultivar name was already confidently assigned. Then, such cultivar could get a working title based on the pomological classification and phenology or if there was no information at all, the designated molecular group was used in place of a cultivar name.

All of these adjustments were necessary to create a high-quality data set. Of the initial 8,354 samples, 170 samples had to be removed from the dataset. The modified dataset consisted of 8,184 samples with consistent pomological and molecular data for further analyses (unpublished, JKI-ZO Dresden-Pillnitz).

#### Creating the most optimal genetic profile per apple cultivar

With the optimized dataset of 8,184 samples, it was aimed to compile unique representing genetic profiles for each apple cultivar. Here, individual cultivars are represented by different numbers of counts of samples with a range from 1 to 73 samples per cultivar. Per individual samples the specific AFL for each marker can differ within the same cultivar. These differentiations are caused by technical variation at the PCR step and subsequent misreads during computational chromatogram analysis which causes shifts in the AFLs^20,21^. A Python script, with assistance of an AI language model, was written to identify the most frequent AFLs per SSR marker position and published at zenodo.org^14,22–25^. This script also returned information for cultivars were manual adjustment was necessary. If no AFL (=0) could be detected for the respective marker in most samples, but there was at least one sample that had an AFL value, the missing value (0) had to be replaced by this value. For cases were no most common AFL could be found due to the heterogeneity in the cultivar samples or deviations due to technical reasons, these specific cultivars had to be checked based on personal evaluation as well. In the following exemplary cases of such adjustments are presented:

**Case 1: Choosing the more heterogeneous option for the genetic profile**. If most samples showed AFLs of e.g. 221:0 and some samples 221:231, the more heterogeneous option (221:231) was chosen as representative AFLs, as long as the difference between the two alleles was more than 1 bp. It is not known if this locus, has a null allele (221:0), is homozygous (221:221) or if the second AFL was not measured (221:231).

**Case 2: Diploid genotypes show more than two AFLs**. In some cases, cultivars known or suggested to be diploid displayed three or more AFLs. For example, the genetic group MD_1521 results in the AFL combination of 109:115:150 but also 115:150 at marker Hi02c07 for the analysed samples. Still the 109:115:150 was chosen as the differences larger than 1 bp. The marker Hi02c07 is also reported to show a multi locus type and this genotype is in line with that^12^. This phenomenon also highlight that the ploidy level cannot be directly inferred from SSR data.

**Case 3: Adjusting AFLs with respect to ploidy level**. For Loci with 3 AFLs in diploid cultivars, the individual AFLs were checked for that loci across the dataset. If an AFL was unique to one genotype, but many other genotypes resulted in an AFL that differed by only 1 bp, the unique AFL was adjusted. This was meant to remove artefacts from the processing of the chromatograms. A special example where the difference in AFL was 2 bp is molecular group MD_0760. Here, manual adjustment of diploid ‘Annie Elizabeth’ (MD_0760) at marker Ch04f10 from 222:224:245 to 224:245 was suggested by ecogenics GmbH (c/o Microsynth AG, Balgach, Switzerland) due to inconsistencies with ploidy level.

The trueness-to-type criterion of the individual samples was compiled for the respective cultivar and scheme of preference was used. If at least one sample per cultivar was assessed with a “1”, the cultivar counted as evaluated with “1” (Table 3). The order for the criteria from preferred to not preferred was “1” > “5” > “2”. For criteria “3” and “4” the most common value was chosen. AS mentioned before “R” was changed to “1” for data format reasons and essentially express the same degree of trueness-to-type.

After these revisions, a finalized dataset of 1,404 apple cultivars with a corresponding unique genetic profile and evaluation of trueness-to-type was compiled and published^1^. Mutation cultivars are masked by their originating cultivar names as they share the same genetic profile. This dataset is valuable due to the intense examination process of the cultivars and therefore high quality and very reliable.

## Data Records

This data descriptor contributes to the transparency and reproducibility of a dataset that is stored in the OpenAgrar (https://www.openagrar.de/content/index.xml) repository and identifiable by 10.5073/20231220-114634-0^1,26^. The dataset (file: “Data_Microsatellite_Broschewitz_231218.xlsx”) represents genetic profiles of apple cultivars of the GFG in form of SSR markers. Additional information on cultivar authenticity as trueness-to-type, international alignment using the Malus UNiQue genotype code (MUNQ)^18,19^ and ploidy level is provided. The data is given in.xlsx-file format and this file has two table sheets. The first table sheet contains the actual data and the second table sheet accommodates concise explanations of the individual columns of table sheet 1 as well as literature references for the SSR markers.

## Technical Validation

Knowledgeable experts performed the pomological assessment. To reduce biases at least two experts had to agree on the cultivar name. Furthermore, the experts were required to provide literature reference that were relevant in the identification process.

The molecular data was provided by ecogenics GmbH (c/o Microsynth AG, Balgach, Switzerland). Experimentally, per 96-well-plate, an isolation control, a PCR control, three multiplex-specific allele ladders and a positive control were included. Technical replicates for four genotypes were performed for projects 2017–2021. During data analysis, the allele fragment length data was adjusted by aligning eight reference genotypes.

Creating consensus genetic profiles per apple cultivar was meant to reduce processing time by automating instead of manual selection. A reciprocal alignment showed that 1,299 (ca. 92.5%) out of 1,404 consensus genotypes are identical to individual genotypes and can therefore be assigned to an actual accession. The remaining 105 consensus genotypes are actually synthetic and optimized out of individual genotypes but could not be allocated to a specific accession.

## Usage Notes

Here, a curated and reliable dataset was created for apple cultivars in Germany. The MUNQ numbers simplify referencing across international databases. Generally, the dataset is well suited for alignment of microsatellite data to infer possible cultivar identities. Highest effort was put in the transparency of authenticity evaluation of the cultivars. The dataset is suited for a wide range of analyses such as parentage, genetic structure or phylogenetic analysis as presented by Broschewitz *et al*.^27^.

As the dataset was curated for usage of the GFG, preferred names of the apple cultivars are given in German language. Possible synonyms can be individually looked up on the website of the GFG (https://www.deutsche-genbank-obst.de/) which is updated regularly. Furthermore, this dataset is biased towards German apple cultivars, as these are inherently the focus of conservation.

Since the genetic profile per cultivar as compiled from several samples and the specific allele fragment length can differ between samples, it is possible that alternative genetic profiles apply.

## Supplementary information

Supplemental TableS1

Supplemental TableS2

## Data Availability

The script for the creation of the consensus genetic fingerprints as well as the script for matching genetic profiles is provided at zenodo.org^25^. These scripts were run in the command line via Anaconda Navigator 2.3.1^23^.Programming was done with Python V 3.10.14 in PyCharm Community V2022.3.2^14,22^. Manual data adjustments and storage was done with Microsoft Excel^28^.
